# Ectoparasites Prevalence in Small Ruminants in and around Sekela, Amhara Regional State, Northwest Ethiopia

**DOI:** 10.1155/2015/216085

**Published:** 2015-04-05

**Authors:** Zewdu Seyoum, Tsegaye Tadesse, Agerie Addisu

**Affiliations:** ^1^Faculty of Veterinary Medicine, University of Gondar, P.O. Box 196, Gondar, Ethiopia; ^2^Amhara National Regional State Bureau of Agriculture, Sekela, Ethiopia; ^3^College of Natural and Computational Sciences, University of Gondar, P.O. Box 196, Gondar, Ethiopia

## Abstract

This study was conducted to determine the prevalence and type of ectoparasites and to identify risk factors associated with ectoparasite infestations in small ruminants in and around Sekela, Northwest Ethiopia. Clinical examination and laboratory analysis were made on 304 sheep and 96 goats. The collected raw data were analyzed using *χ* ^2^-test. Out of the 400 sampled animals, 182 (45.5%) were infested with one or more ectoparasites. The prevalent ectoparasites observed were lice, ticks, *Ctenocephalides* species, *Melophagus ovinus*, and *Demodex* species. The infestation rates of ectoparasites with age and sex were significantly varied (*P* < 0.05) in sheep but not in goats (*P* > 0.05). Body condition score was not significantly associated (*P* > 0.05) with ectoparasites infestation in both sheep and goats. In our attempt, only two cases due to *Demodex* species were recorded in sheep. In conclusion, the prevalence of ectoparasites in the present study was high and this could affect the wellbeing and productivity of small ruminants. Therefore, to reduce ectoparasites prevalence and impact on the productivity and health status, planning of integrated control measures with sustainable veterinary services aiming at creating awareness about the importance and control of ectoparasites for livestock owners is required.

## 1. Introduction

Ethiopia with its greatest variation in climate and topography possesses one of the largest small ruminant populations in the world, which is kept extensively mostly by small holder farmers and adjacent to crop production [1, 2]. Small ruminants represent an important segment of the Ethiopian livestock system. They are important sources of income for the agricultural communities and are one of the country major sources of foreign currency through skin and meat export and are among important sources of animal protein, providing 35% of meat and 14% of milk consumption. The national small ruminant population is estimated to be 63 million heads, which are raised in different agroecological regions of the country [2]. However, the contribution from this huge population to food production and export income is far below the expected potential. This would be due to the compound effects of several factors among which is ectoparasitism [1, 3].

Infestation by ectoparasites could lead to considerable economic losses to farmers due to loss of productivity, mortality, and skin diseases. Ectoparasites including lice, sheep keds, ticks, fleas, and mange mites are reported to cause a wide range of health problems such as mechanical tissue damage, irritation, inflammation, hypersensitivity, abscesses, weight loss, lameness, anaemia, and in severe cases death of infested animals with the consequent socioeconomic implications [4–7]. In addition, ectoparasite infestations could induce great economic losses due to reduction of wool quality, meat and milk yield, and losses as a result of culling and related with cost of treatment and prevention of the problem. They are also responsible for great preslaughter skin defects, resulting in downgrading and rejection of small ruminant skins [8, 9]. According to tanneries reports, skin defects due to ectoparasite effects cause 35% of sheep and 56% of goat skin rejections in Ethiopia [10]. Moreover, ectoparasites are known to have zoonotic importance and be capable of transmitting several types of disease pathogens from animals to animals and from animals to human due to their blood sucking habit [7, 9]. All these established facts imply that ectoparasites cause serious economic losses to the farmer, the tanning industries, and the country as a whole [8, 9].

Despite these important consequences to animals and human beings, the prevalence and magnitude of ectoparasite infestation in small ruminants have not been assessed in and around Sekela, Northwest Ethiopia. Therefore, information on prevalence, distribution, and potential risk factors of ectoparasites of small ruminants is significant because the outcome could be used to make objective decisions on control strategies. The finding would also help in formulating strategies to meet the current shortfall of animal product created by the rapidly increasing human population. Hence, the present study was planned (1) to identify ectoparasites that parasitize small ruminants in and around Sekela area, (2) to determine the prevalence of ectoparasites infestation in relation to risk factors such as species, age, sex, and body condition score of study animals, and (3) to recommend suitable preventive and control strategies.

## 2. Materials and Methods

### 2.1. Study Area

The present study was conducted on ectoparasites of small ruminants in and around Sekela, Northwest Ethiopia, from October, 2013 to April, 2014. Sekela is located between 10°59.25′N latitude and 36°55.30′E longitude, in the northwest Ethiopia, at 460 km from Addis Ababa. Topographically, it has an elevation of 1500–3200 m.a.s.l. The area mean annual rainfall is 1700 mm and the mean annual temperature is 18°C. The farming system in the area is characterized as mixed crop-livestock production systems. The livestock in the study area is traditionally managed under extensive production system. According to CSA [11] census result, the study area has 73,170 cattle, 12,264 equines, 152,545 small ruminants, and 26,725 chickens.

### 2.2. Study Animals and Clinical Examination

In our study, a total of 400 small ruminants (304 sheep and 96 goats) of different age groups, both sexes and of local breeds coming to the Sekela district Veterinary Clinic for veterinary services, were examined for the presence of lice, fleas, ticks, mange mites, and skin lesions. During sampling, history, species, age, sex, and body condition of each animal were recorded. The animals were grouped into two age categories, as young (up to one year) and adult (older than one year) as described by [12, 13]. Age determination was made using owner's information and by dentition. Body condition scores were determined following the procedures documented by Steele [12] and ESGPIP [13] for sampled animals as poor, medium, and good classes following 1 up to 5 grading system. However, our study was conducted during dry season of the year and the body condition score of most of studied animals was very poor and the animals were emaciated. Thus, differentiating among medium and poor conditioned animals was difficult. Therefore, the authors of this paper preferred to assign the studied animals to poor and good body condition score groups. A poor body condition score was given for animals which were extremely thin, having prominent spinous and transverse processes into which a finger could be easily pushed, and had less depth of loin muscle. A good body condition score was given for animals when the spinous and transverse processes were smooth, rounded, and well covered and with full loin muscle [6, 13].

Clinical inspection of each sampled animal was performed visually and by multiple fleece partings, followed by physical examination of skin, inspection, and palpation of the skin across all parts of the animal for the presence of parasites and gross lesions indicating the clinical form of infestation by ectoparasites. Animals found with ectoparasites were considered as positive.

### 2.3. Ectoparasite Collection and Identification

After proper restraining, representative specimens were collected from infested and diseased animals. Ectoparasites (sheep keds, ticks, lice, and fleas) either encountered on the skin surface or attached to the hair were collected manually from their sites of attachment. The ticks were removed from the host skins whilst retaining their mouth parts for identification using thumb forceps. A coat brushing technique was applied to collect lice from host skin. Then the collected samples were placed in labelled universal bottles containing 70% ethanol and taken to the Parasitology Section Entomology Laboratory of the Bahir Dar Regional Animal Health Diagnostic and Investigation Centre located in Bahir Dar town. In the laboratory, the ectoparasites were identified with the basis of their morphological structure using the recommendations of Urquhart et al. [14] and Wall and Shearer [15]. Further identification at species level was conducted using a stereomicroscope according to their key morphological structures using Walker et al. [16] suggestions for ticks and Urquhart et al. [14] and Wall and Shearer [15] for lice and fleas.

In addition, skin scrapings for mange mites were collected from clinically suspected animals. This was made by clipping the hair around affected areas using scissors, scraping the edges of the lesion with scalpel blades [14] until capillary blood oozing was evident. The scraped materials were transferred to a container containing 10% formalin and were taken for laboratory examination. Then in the laboratory, a few drops of 10% potassium hydroxide were added to the specimen, allowed to stand for 30 minutes, and examined under a light microscope at 40x magnification [3, 14]. The mange mites were identified with the morphological keys of Urquhart et al. [14] and Wall and Shearer [15].

### 2.4. Data Management and Analysis

A Microsoft Excel spread sheet was used for raw data management. Statistical software SPSS version 17 was used for data analysis. Descriptive statistics such as percentage were used to summarize the proportions of infested and noninfested sampled animals. The association with different risk factors (age, sex, body condition, and species of animals) on the prevalence and distribution of ectoparasites was analyzed using *χ* ^2^-test. The differences were considered as significant when *P* < 0.05 at 95% confidence intervals.

## 3. Results

### 3.1. Overall Prevalence of Ectoparasites

The overall prevalence of ectoparasites (45.5%) was recorded on examined animals. From 304 sheep and 96 goats examined for ectoparasites, 145 (47.7%) sheep and 37 (38.5%) goats were found to be infested with one or more ectoparasites. Tick infestation (22.7%),* Linognathus ovillus* (14.2%),* Ctenocephalides* spp. (10.52%),* Melophagus ovinus* (9.2%),* Bovicola ovinus* (8.9%), and* Demodex* spp. (0.66%) were the identified ectoparasites in sheep. Similarly, the identified ectoparasites on goats include* Ctenocephalides* spp. (17.7%),* Linognathus stenopsis* (17.7%),* Melophagus ovinus* (12.5%), and tick infestation (11.5%). The tick species identified in sheep were* Rhipicephalus evertisi* (12.5%),* Boophilus decoloratus* (6.3%),* Amblyomma variegatum* (2.3%), and* Hyalomma marginatum* (1.6%) while in goats* R. evertisi* (6.3%),* B. decoloratus* (4.2%), and* A. variegatum* (1.04%) were identified (Table 1).

### 3.2. Species-Wise Prevalence of Ectoparasites

**Figure 1:**
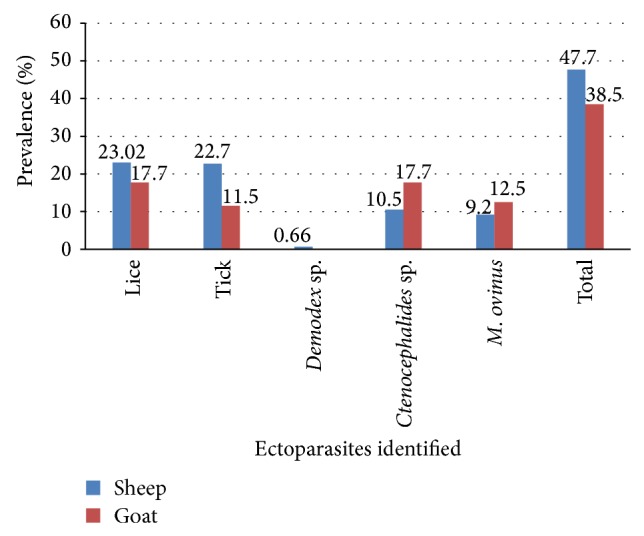
Species-wise prevalence of ectoparasites in sheep and goats.

The overall prevalence of ectoparasite infestation in sheep (47.7%) and goats (38.5%) was not significantly varied (*χ* ^2^ = 2.466 and *P* = 0.116). However, the prevalence of tick infestation in sheep (22.7%) was significantly more prevalent than in goats (11.45%) (*χ* ^2^ = 5.76 and *P* = 0.016). Statistically significant differences were never recorded (*P* > 0.05) in the prevalence of lice,* Ctenocephalides* species, and* M. ovinus* between sheep and goats. In our study, only two cases (0.66%) due to* Demodex* species were identified in sheep, but no demodectic cases were recorded in goats (Figure 1).

### 3.3. Sex, Age, and Body Condition Score-Wise Prevalence of Ectoparasites

As indicated in Table 2, from factors considered, sex and age in sheep population were found to be risk factors for infestation with ectoparasites. Higher ectoparasite prevalence (*χ* ^2^ = 13.577 and *P* = 0.000) was observed in male sheep (64.4%) than female sheep (41%). The prevalence of lice in female sheep (26.3%) was significantly (*χ* ^2^ = 4.494 and *P* = 0.034) higher than in males (14.9%) while the prevalence of* M. ovinus* and ticks infestation was significantly (*P* < 0.05) higher in male sheep than females. Similarly, young group of sheep appeared to be more frequently infested with* M. ovinus* and ticks (*P* < 0.05) than adult group of sheep. Moreover, the rate of infestations of sheep with* Ctenocephalides* spp. and* M. ovinus* was significantly higher in poor than in good body condition score sheep (*P* < 0.05) (Table 2). However, the prevalence of* Ctenocephalides* spp. was not significantly (>0.05) varied among sex and age groups of sheep. Similarly, lice infestation was not significantly associated with age and body condition score of sheep. Moreover, tick infestation prevalence was never associated with body condition score of sheep.

In goats, the overall prevalence of ectoparasite infestation was not associated with the factors that are deemed to be risk factors. However,* Ctenocephalides* spp. and* M. ovinus* infestation was significantly (*P* < 0.05) prevalent in young goats than adults and in poor body condition score goats than good condition score goats (Table 3). Other recorded ectoparasite infestation with lice and tick did not show any association with the considered factors (age, sex, and body condition score).

## 4. Discussion

In the present attempt, we determined the prevalence of ectoparasite infestation in small ruminants brought to Sekela Veterinary Clinic using clinical examination. It is therefore noted that the prevalence of ectoparasites estimates provided here may have some limitations as samples from clinical case may not always represent the reference population where animals are drawn. This is because animals brought for clinical services are those that are often clinically diseased or stressed ones. So, there is a possibility that more positive cases are observed, resulting in overestimating the actual ectoparasite burden. In spite of these limitations, clinical survey data may be used to estimate the ectoparasite burden because of easy feasibility of conducting clinical surveys compared to field surveys based on random study designs. In addition, clinical survey data may provide opportunities for designing intervention strategies by timely diagnosis and treating animals infested with ectoparasites that influence the quality of skin and productivity of infested animals.

The overall prevalence of ectoparasite infestation in the present study was found to be 45.5%. This suggested the great importance of ectoparasites in small ruminants of the study area. This finding is in line with previous reports from Ethiopia [3, 17] and elsewhere in the world [18, 19]. Similarly, our finding coincides with the reports on Yacob et al. [20]; Mulugeta et al. [21]; and Tesfaye et al. [22]. These higher infestation rates might be attributed to various important factors including favourable climatic factors, malnutrition especially during long dry season, poor husbandry system, poor awareness of farmers to the effects of ectoparasites, and inadequate animal health services in the study area [3, 19, 23].

In this attempt, the overall infestation rate of ectoparasites was not significantly varied among sheep and goats. This finding suggested that sheep and goats are equally susceptible to the identified ectoparasites. This contradicts with the findings of Yacob et al. [20], Fentahun et al. [24], and Tesfaye et al. [22] in Ethiopia. Similarly, Edoga [25] also reported host differences in susceptibility to ectoparasites in Nigeria.

The overall prevalence of lice infestation in sheep and goats was found to be 23.02% and 17.7%, respectively. Lice have been considered as one of the responsible parasites for skin rejection at tanneries in Ethiopia [8, 9, 21] due to a skin defect as a result of itching leading to scratching and rubbing due to feeding behaviour of lice. Moreover, lice infestation could be responsible for production loss, irritation, and disease transmission [15]. The lice infestation rate observed in this study is by far higher than previous observations from different parts of Ethiopia by Haffize [26], Beyecha et al. [6], and Tesfaye et al. [22]. In contrast, Sertse and Wossene [17], Wall and Shearer [15], and Kumsa et al. [3] observed higher prevalence of lice infestation in sheep and goats. This discrepancy might be attributed to differences in agroclimate that favour the biology of lice, population density, study method and period, the husbandry system, and health services in the study area. The occurrence of lice infestation could indicate some other basic concerns such as malnutrition and chronic diseases [14, 15, 21]. Pugh [27] also stated lice infestation could be higher in emaciated animals that suffer from malnutrition and parasitic diseases.

The overall prevalence of tick infestation was higher in young sheep than in adult animals. This is consistent with the reports on Urquhart et al. [14] that reported higher tick infestation rates for young and poor body condition than adults and good body condition animals. This may be as a result of incapability of young and debilitating or emaciated animals to groom and leak themselves and presence of weak defence mechanism in these groups of animals [14]. Lower tick infestation prevalence in this study was observed in sheep and goats in comparison with Abunna et al. [28] who reported higher prevalence in sheep (87.5%) and goat (89.9%) in Miesso district, Ethiopia. This could be due to an ectoparasite control campaign that has been practiced for ectoparasites control for three consecutive years from 2008 to 2010 in Amhara Regional State, Ethiopia. Moreover, this may be allied with variations in climatic factors and geographical locations. Climatic factors (environmental temperature and relative humidity) are considered the major ecological structure for the reproduction and growth of tick populations [29].

*Ctenocephalides* species were the third commonly examined ectoparasite next to lice and tick in small ruminants in the present study. There were significant variations in the prevalence of flea infestation with age and body condition of both sheep and goats. This observation coincides with previous reports of Yacob et al. [20], Mulugeta et al. [21], Bekele et al. [30], Beyecha et al. [6], and Tesfaye et al. [22] in Ethiopia. But no significant association was encountered with prevalence of fleas and sex of study animals in the present observation. This agrees with the reports on Yacob et al. [20] and Fentahun et al. [24] from Ethiopia.

Infestation with* M. Ovinus* leads to irritation that results in skin and fleece damage from animal biting and rubbing and staining of wool by faeces of the ked [10].* Melophagus ovinus* was the other widely observed ectoparasite in sheep (9.2%) and goats (12.5%) in our study. This observation is comparable with the report on Kassaye and Kebede [31]. However, the present result disagrees with the reports on Mulugeta et al. [21] and Fentahun et al. [24], which showed 19.1% and 20.1% infestation rates of* M. ovinus* elsewhere in Ethiopia, respectively. This might be due to differences in environmental factors of the study sites and study period. According to Pugh [27],* M. ovinus* is highly populated or attains peak level during the wet season, but the present study was conducted in the long dry season which would contribute to a lower rate of observation. Analysis of seasonal densities of sheep ked by Legg et al. [32] also indicated that sheep ked populations are mainly seen in colder, wetter areas and the infestation may be lost when the sheep are moved to hot and dry areas. According to Radostatits et al. [33] in the hot-humid tropics the parasite is restricted to cooler highlands and infestations may be lost when sheep are moved to hot dry areas.

In conclusion, the present study identifies lice, ticks,* Ctenocephalides* species, and* M. ovinus* to be the major ectoparasites of small ruminants. These ectoparasites have been identified as the major causes of sheep and goat production constraints and quality deteriorations of skin in Ethiopia. Therefore, the growing threat from ectoparasites on overall sheep and goats' productivity and tanning industry in Ethiopia warrants urgent control intervention. Hence, to manage the effects of ectoparasites in small ruminant populations it would be valuable to implement effective extension system and programs that could lift up community awareness on management of animals, effect of ectoparasites, and practicable strategic control measures (restriction of animal movement from endemic areas and chemical applications) with full cooperation of farmers and responsible bodies in the area.

## Figures and Tables

**Table 1:** Prevalence (%) of ectoparasites observed in small ruminants coming to Sekela Veterinary Clinic.

Ectoparasites	Sheep (*n* = 304)		Goats (*n* = 96)	
	Infected	Prevalence (%)	Infected	Prevalence (%)
Lice				
*Linognathus ovillus *	43	14.2	—	—
*Bovicola ovinus *	27	8.9	—	—
*Linognathus stenopsis *	—	—	17	17.7
Tick				
*Rhipicephalus evertisi *	38	12.5	6	6.3
*Boophilus decoloratus *	19	6.3	4	4.2
*Amblyomma variegatum *	7	2.3	1	1.04
*Hyalomma marginatum *	5	1.6	—	—
*Melophagus ovinus *	28	9.2	12	12.5
*Ctenocephalides* species	32	10.5	17	17.7
*Demodex* species	2	0.66	—	—

Overall prevalence	145	47.7	37	38.5

**Table 2:** Prevalence (%) of ectoparasites in sheep (*n* = 304) with sex, age, and body condition.

Ectoparasites	Sex		*P* value	Age		*P* value	Body condition		*P* value
	Male (*n* = 87)	Female (*n* = 217)		Young (*n* = 26)	Adult (*n* = 278)		Poor (*n* = 253)	Good (*n* = 51)	
Lice	13 (14.9%)	57 (26.3%)	0.034	7 (26.9%)	63 (22.7%)	>0.05	63 (24.5%)	7 (13.7%)	>0.05
Tick	29 (33.3%)	40 (18.4%)	<0.01	15 (57.7%)	54 (19.4%)	<0.001	62 (24.5%)	7 (13.7%)	>0.05
*Demodex *spp.	1 (1.1%)	1 (0.5%)	>0.05	1 (3.8%)	1 (0.4%)	>0.05	2 (0.8%)	0 (0.0%)	>0.05
*Ctenocephalides* spp.	12 (13.8%)	20 (9.2%)	>0.05	3 (11.5%)	29 (10.4%)	>0.05	32 (12.6%)	0 (0.0%)	<0.01
*Melophagus ovinus *	13 (14.9%)	15 (6.9%)	0.029	9 (34.6%)	19 (6.8%)	<0.001	28 (11.1%)	0 (0.0%)	<0.01

Overall prevalence	56 (64.4%)	89 (41%)	<0.001	18 (69.2%)	127 (45.7%)	0.022	122 (48.2%)	23 (45.1%)	>0.05

**Table 3:** Prevalence (%) of ectoparasites in goats (*n* = 96) with sex, age, and body condition.

Ectoparasites	Sex		*P* value	Age		*P* value	Body condition		*P* value
	Male (*n* = 33)	Female (*n* = 63)		Young (*n* = 24)	Adult (*n* = 72)		Poor (*n* = 67)	Good (*n* = 29)	
Lice	4 (12.1%)	13 (20.6%)	>0.05	3 (12.5%)	14 (19.4%)	>0.05	10 (14.9%)	7 (24.1%)	>0.05
Tick	5 (15.2%)	6 (9.5%)	>0.05	2 (8.3%)	9 (12.5%)	>0.05	9 (13.4%)	2 (6.9%)	>0.05
*Demodex *spp.	—	—	—	—	—	—	—	—	—
*Ctenocephalides* spp.	6 (18.2%)	11 (17.5%)	>0.05	11 (45.8%)	6 (8.3%)	<0.001	17 (25.4%)	0 (0.0%)	<0.001
*Melophagus ovinus *	4 (12.1%)	8 (12.7%)	>0.05	7 (29.2%)	5 (6.9%)	<0.01	12 (17.9%)	0 (0.0%)	0.016

Overall prevalence	16 (48.5%)	21 (33.3%)	>0.05	9 (37.5%)	28 (38.9%)	>0.05	28 (41.8%)	9 (31%)	>0.05
